# Research on the Effects of Drying Temperature for the Detection of Soil Nitrogen by Near-Infrared Spectroscopy

**DOI:** 10.3390/molecules28186507

**Published:** 2023-09-07

**Authors:** Ling Zhou, Jiangjun Yao, Honggang Xu, Yahui Zhang, Pengcheng Nie

**Affiliations:** 1 College of Information Engineering, Tarim University, 1188 Junken Avenue, Alar 843300, China; 2 Key Laboratory of Tarim Oasis Agriculture, Ministry of Education, Tarim University, 1188 Junken Avenue, Alar 843300, China; 3 College of Biosystems Engineering and Food Science, Zhejiang University, 866 Yuhangtang Road, Hangzhou 310058, China; 4 Key Laboratory of Spectroscopy Sensing, Ministry of Agriculture and Rural Affairs, Hangzhou 310058, China

**Keywords:** nitrate–nitrogen, near-infrared sensor, drying temperature, PLS, SVM, ANN

## Abstract

Nitrogen nitrates play a significant role in the soil’s nutrient cycle, and near-infrared spectroscopy can efficiently and accurately detect the content of nitrate–nitrogen in the soil. Accordingly, it can provide a scientific basis for soil improvement and agricultural productivity by deeply examining the cycle and transformation pattern of nutrients in the soil. To investigate the impact of drying temperature on NIR soil nitrogen detection, soil samples with different N concentrations were dried at temperatures of 50 °C, 65 °C, 80 °C, and 95 °C, respectively. Additionally, soil samples naturally air-dried at room temperature (25 °C) were used as a control group. Different drying times were modified based on the drying temperature to completely eliminate the impact of moisture. Following data collection with an NIR spectrometer, the best preprocessing method was chosen to handle the raw data. Based on the feature bands chosen by the RFFS, CARS, and SPA methods, two linear models, PLSR and SVM, and a nonlinear ANN model were then established for analysis and comparison. It was found that the drying temperature had a great effect on the detection of soil nitrogen by near-infrared spectroscopy. In the meantime, the SPA-ANN model simultaneously yielded the best and most stable accuracy, with $R_{c}^{2}$ = 0.998, $R_{p}^{2}$ = 0.989, RMSEC = 0.178 g/kg, and RMSEP = 0.257 g/kg. The results showed that NIR spectroscopy had the least effect and the highest accuracy in detecting nitrogen at 80 °C soil drying temperature. This work provides a theoretical foundation for agricultural production in the future.

## 1. Introduction

In order for plants to grow and flourish, soil is crucial to agricultural production. The rate of development, yield, and quality of plants are all influenced by the soil, as it provides the water, oxygen, nutrients, and strength [1]. Therefore, it is of great importance to maintain good soil quality, including maintaining and improving soil fertility, structure, moisture, pH, and biodiversity. Moreover, among the various nutrients in soil, nitrogen is not only the basis for the formation of plant proteins, chlorophyll, nucleic acids, enzymes, and other biological molecules but also one of the important elements required for plant growth [2]. In particular, nitrate–nitrogen is an essential component of nutrient cycling in soils. Excessive levels of nitrate–nitrogen can lead to environmental problems such as soil acidification, water eutrophication, and groundwater contamination [3]. Moreover, nitrate–nitrogen can be converted into nitrite in plants, and nitrite easily combines with protein to form the carcinogenic substances nitrosamines, which are harmful to human health [4]. Hence, a close study of soil nitrogen can determine the degree of soil fertility and offer a scientific basis for plant cultivation, which can provide guiding suggestions for precise fertilization. It also facilitates the protection of the environment and prevents overfertilization and contamination of the soil [5,6,7,8,9]. Accordingly, the rapid and accurate acquisition of nitrogen content is of great significance to agricultural production and environmental protection [10].

However, the conventional soil nitrogen testing methods, such as Kjeldahl digestion, dry combustion, and ion-sensing electrodes, are usually destructive, costly, and time-consuming [11]. Moreover, since this is affected by the technical level of the operator and so on, the recognition results will have a certain error and cannot meet the application of real-time accurate detection scenarios [12,13]. Due to its high efficiency and nondestructive accuracy, spectroscopy has been widely used to detect soil nitrogen in recent years [14]. Near-infrared spectroscopy, which has developed into a mature and major chemical analysis technique, has especially been applied [15]. For soil, the spectral information associated with most of the organic radical groups containing hydrogen was located in the near-infrared region [16,17]. Due to its nondestructive, nonpolluting, and quick detection process, many scholars have performed research on nitrogen detection using this technique. In the first place, the use of different algorithms to select the feature bands on spectral modeling was investigated. Zhang et al. [12] developed two methods for identifying sensitive bands of soil TN content. The results showed that the eight sensitive bands selected by the combined ant colony algorithm and mutual information algorithm (ACO-MI) method had a good mechanism, generality, and predictive ability in estimating the total soil nitrogen content. Wang et al. [18] used a public dataset called LUCAS Soil and proposed a deep-learning-based wavelength screening method for STN features. With the three other prediction methods, namely ordinary least squares estimation (OLSE), random forest (RF), and extreme learning machine (ELM) modeling, the results indicated that the ELM model using STN feature wavelengths performs better. Xiao et al. [19] selected the characteristic bands of loess, calcareous soil, black soil, and laterite by using competitive adaptive weighted sampling (CARS) and double least squares and modeled them based on partial least squares (PLS), inverse interval partial least squares (BIPS), and the backpropagation neural network (BPNN). The results showed that determining the sensitive bands for different soil types can largely improve the efficiency of nitrogen detection. Secondly, the soil water content also has a great influence on the spectral detection of nitrogen. Wang et al. [20] found that for the detection of soil nitrogen, dry soil had a better performance than moist soil. He et al. [21] treated the soil samples with drying times ranging from 1 h to 8 h, measured the corresponding moisture content at hourly intervals, and modeled the predictions using partial least squares (PLS) and uninformative variable elimination (UVE). The experiments suggested that the highest accuracy for the NIR detection of soil nitrogen was achieved when the soil moisture content was 1.03 percent. An et al. [22] proposed a moisture absorption correction method (PMAI), which normalized the raw spectral data to standard spectral data and then used backpropagation network modeling to predict the total nitrogen content. The results demonstrated that this method can effectively eliminate the interference of the soil water content on the prediction of the total soil nitrogen content. Zhou et al. [23] proposed a method of moisture absorption correction index that eliminated soil moisture interference. A new coupled soil moisture and particle size elimination method for predicting the total soil nitrogen from discrete near-infrared spectral band data was then created by combining it with a particle size correction coefficient (PSCI). In addition, the temperature can also affect the detection of soil nitrogen by using NIR spectroscopy [24]. Particularly, the drying temperature had an effect on water removal and urease activity [25]. The effect of soil water content on the determination of soil nitrogen via NIR spectroscopy was more studied, while the effect of drying temperature on the determination of nitrogen via NIR spectroscopy was less studied. Coarse samples were dried at 20 °C for 48 h and used to predict carbon and nitrogen content by using visible near-infrared spectroscopy and to detect their potential mineralization in nonhomogeneous soil samples, using comparative methods [26]. Flat-dry samples were dried at 35 °C for 12 h to explore the effects of pretreatment and standardized rewetting of soil samples [27]. Nie et al. [28] studied the effect of drying black, loess, and calcareous soils at different temperatures on NIR detection of soil nitrogen, and the results showed that different soil types have different suitable drying temperatures. In general, drying is usually used to remove soil moisture and is mostly focused on the detection of soil nitrogen. However, to our knowledge, there are few optimal drying temperatures considering nitrate–nitrogen content in the present study, and the mechanism of its influence is not clear. Based on this, we would like to fill the research gaps in this area and hope to further enhance the possibility of soil nitrogen detection precision by NIR.

In accordance with the existing studies, this study focused on the effect of soil-drying temperature on the detection of soil nitrogen by using NIR sensors. After analyzing the raw soil spectra, preprocessing methods, and characteristic bands, an optimal soil-drying temperature was derived, and higher prediction accuracy was achieved, with the aim of improving the theoretical and experimental guidelines for achieving precise fertilization.

## 2. Results

### 2.1. Soil Original NIR Spectral Characterization

**Figure 1:**
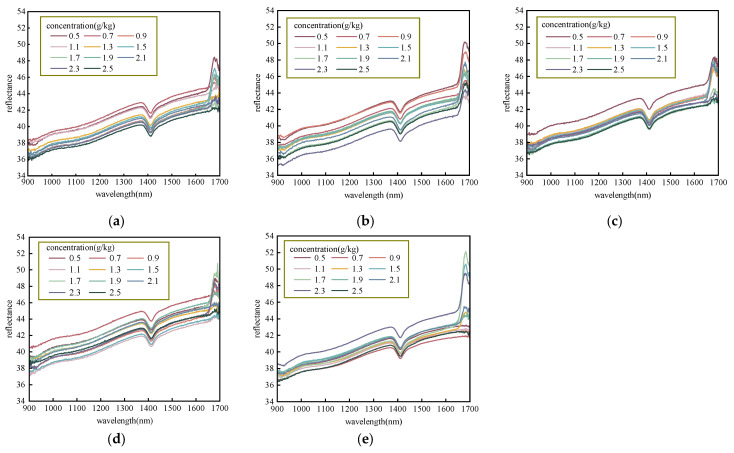
Average NIR reflectance spectra of soils (**a**) placed at 25 °C, (**b**) dried at 50 °C, (**c**) dried at 65 °C, (**d**) dried at 80 °C, and (**e**) dried at 95 °C.

In this experiment, the spectral information of soil samples with different N concentrations at five drying temperatures was collected using a near-infrared sensor. Referring to Figure 1, the horizontal coordinate of the curve is the wavelength, and the vertical coordinate of the curve is the average spectral reflectance. The NIR reflectance curves for soil air-dried at 25 °C, 50 °C, 65 °C, 80 °C, and 95 °C are shown in Figure 1a–e. Each image reflects the variation in the reflectance spectra with the wavelengths for soil samples at different drying temperatures.

The NIR spectra of different soils varied, but the overall trends were similar. It can be seen that the spectral reflectance vibration in the 900–930 nm and 1680–1700 nm bands is severe, probably because the NIR sensor has more spectral information overlap and noise at the edge of the acquisition band [29]. In addition, all spectra have a maximum absorption peak near 1400 nm, which corresponds to the second overtone of the N-H bond [20].

The effect of drying temperature on the spectrum shows a certain degree of negative correlation with the reflectance intensity. As the drying temperature increases from 25 °C to 80 °C, there was an overall increase in the reflection peak near 1680 nm This indicates that as the soil-drying temperature increases, the spectral absorptivity decreases considerably, and, thus, the reflectance increases. However, when the drying temperature reaches 95 °C, the spectral reflectance near 1400 nm decreases to within the range of 38 to 42. This, in turn, suggests that the reflection intensity of the spectrum decreases when the drying temperature is too high, which similarly took place in Nie et al.’s research [24]. At the same time, the reflectance curve generally becomes smoother and more concentrated as the drying temperature increases. The soil spectral curve is the most curved at 25 °C air-dried placed at room temperature, and the curve becomes smoother and smoother from 50 °C to 65 °C to 80 °C, while the spectral curve is the smoothest and flattest at 95 °C. The reason for this is that when the soil was placed at 25 °C to air-dry, the soil was affected by the external environment, and the moisture did not dry completely since the NIR spectrum is very sensitive to the absorption of moisture. Although the high temperature completely eliminates the effect of moisture, it perhaps lost some valuable information. For instance, nitrates are unstable at high temperatures and perhaps volatilize in minor amounts.

When the drying temperature is 80 °C, it can be seen that the effect of the drying temperature on soil nitrogen detection in the NIR band is reduced to a relatively low level, and, at this point, the spectral reflectance curves of soil nitrogen are neither too concentrated nor too dispersed. The results show that the gradient of the reflectance curve became more pronounced and better detected with the increasing soil nitrogen concentration under such dry conditions. After the analysis based on the raw spectra, further studies will be performed regarding the data processing.

### 2.2. Full-Band Data Analysis

**Figure 2:**
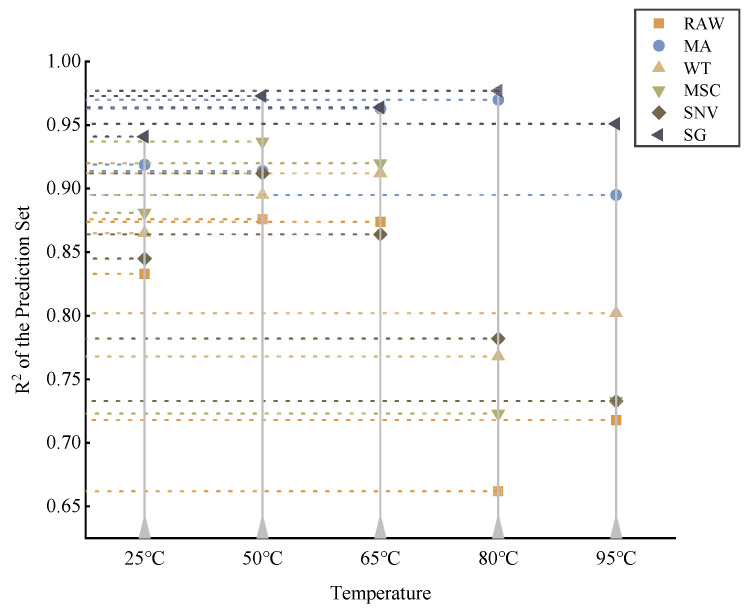
The determination coefficients of the partial least squares (PLS) method after different spectral pretreatments.

With soil spectral reflectance as the independent variable and nitrogen concentration as the dependent variable, the soil samples were divided into training and prediction sets in the ratio of 7:3, and then the original spectra and the other five pretreatment spectra were modeled and analyzed by using PLS, respectively. The predicted results using different pretreatment methods at different drying temperatures are given in Table 1 and Figure 2.

It can be seen that the effect of modeling with PLS after pretreatment from using different methods at different temperatures is not exactly the same. The effect of each pretreatment algorithm does not differ much at low and medium temperatures, while the effect is very unstable at high temperatures, especially at 80 °C and 95 °C. The most obvious performance is at 80 °C and 95 °C. This shows that high drying temperatures are not conducive to the detection of soil nitrogen, although it completely eliminates the moisture effect.

Although the effect of each pretreatment method varies for different temperatures, it is certain that the $R^{2}$ of the raw unpreprocessed spectral information is essentially the lowest regardless of the temperature, indicating that some preprocessing of the spectral data helps to improve the modeling accuracy. After a comprehensive comparison, the best lifting results were obtained with the SG algorithm for all temperatures, according to Table 1. It mathematically builds a polynomial regression to fit the curve and estimate its value at the center of a window of approximation. The algorithm preserves essential features and the data trends, while allowing for simple and fast denoising [30]. This, in part, makes it the best preprocessing method in this study. Consequently, the band selection would be based on the SG preprocessed data.

### 2.3. Feature Wavelength Selection

The full-band-based NIR spectral dataset contains 400 wavelengths of data, which make up a large amount of data, with lots of redundant information unrelated to the spectral response of soil N. Therefore, random forest (RFFS), competitive adaptive reweighted sampling (CARS), and continuous projection algorithm (SPA) feature wavelength selection algorithms were used to find the feature wavelengths associated with the soil nitrogen content. The maximum number of selected variables was set at 35, and wavelength variables were selected from 400 spectral variables based on the minimum error. The number of characteristic wavelengths and bands selected from the five datasets are shown in Table 2.

It is obvious that the number of variables in each dataset was greatly reduced after the selection of feature wavelengths. The number of wavelengths selected by RFFS ranged from 11 to 31, the number of wavelengths selected by CARS ranged from 24 to 33, and the number of wavelengths selected by SPA ranged from 8 to 18. For each temperature dataset, SPA selected fewer feature wavelengths than CARS and RFFS selected, as shown in Table 2.

**Figure 3:**
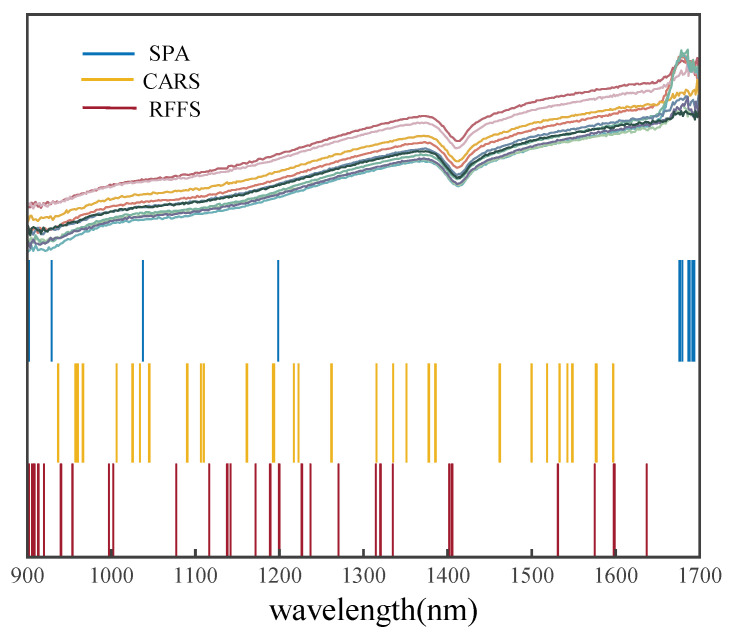
Screening results of each algorithm for the average spectrum of soil dried at 80 °C.

To show the differences in the positions of the feature wavelengths selected by the algorithms more graphically, Figure 3 shows the results of the algorithms filtering the feature parameters for the average soil spectra at 80 °C, and the RFFS, CARS, and SPA methods are marked with different colored vertical lines in the figure. The different algorithms at the same drying temperature selected a different number of variations and wavelength bands, indicating that the drying temperature had a great influence on the wavelength variables selected by the algorithm. According to Figure 3, it is evident that the feature wavelengths selected by CARS and RFFS basically covered the locations where these differences occur, and there is a certain degree of overlap between the sites. However, the SPA, although less selective than these in both, covered these bands with large fluctuations in the spectral curve.

Therefore, the characteristic wavelengths chosen by these three methods were closely related to the nitrogen content in the soil. However, in the previous literature [21,29], the characteristic wavelengths of soil N were rarely selected for analysis. Even when a characteristic wavelength selection algorithm was used, the location of its bands was not specifically or visually analyzed. To demonstrate whether the selected wavelengths are reliable, a further analysis was performed using machine-learning and deep-learning modeling.

### 2.4. Prediction Model and Analysis of Soil Nitrogen Content under Different Drying Temperatures

In this study, PLS, SVM, and ANN models for predicting soil nitrogen content were developed based on the feature bands selected by the above three algorithms, respectively. A total of 132 samples were divided into two datasets by using the holdout cross-validation method; 92 of the samples were used as the training dataset, and the remaining 40 samples were used as the validation dataset. Furthermore, the modeling results are plotted as a 1:1 relationship diagram, making it easy to visualize the fitting effect of each model.

#### 2.4.1. Partial Least Squares Modeling

**Figure 4:**
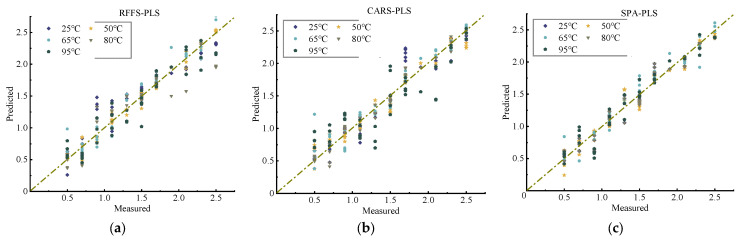
Relationship between actual and predicted values of soil N content based on the PLS model: (**a**) RFFS-PLS, (**b**) CARS-PLS, and (**c**) SPA-PLS.

The results of the PLS model developed by the automatic adjustment of optimal parameters method are shown in Figure 4 and Table 3.

According to Figure 4, it is clear that the SPA-based PLS model fits a little better at all five temperatures. However, comparing RMSEP and RPD according to Table 3, it can be found that the performance of the CARS-based PLS model will be better and more stable when the two $R^{2}$ values are similar. In this case, the $R_{c}^{2}$ at each temperature is above 0.97, the $R_{p}^{2}$ is above 0.95, and the RPD reach 2.170 at 80 °C. The results show that the soil nitrogen is significantly correlated with the selected sensitive bands. This suggests that the accuracy of training and prediction is acceptable when we conduct qualitative studies of soil nitrogen, for example, when only a rough estimate of the nitrogen content in the soil is required. Overall, in terms of PLS modeling, the best prediction is based on the feature bands selected by the CARS algorithm.

#### 2.4.2. Prediction Model of Soil Nitrogen Content Based on Support Vector Machine

**Figure 5:**
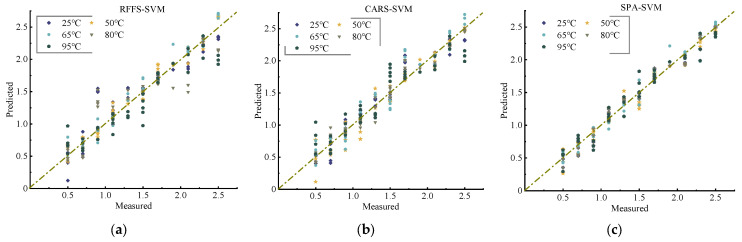
Relationship between actual and predicted values of soil N content based on SVM model: (**a**) RFFS-SVM, (**b**) CARS-SVM, and (**c**) SPA-SVM.

The results based on the developed support vector machine prediction model for soil nitrogen are shown in Figure 5 and Table 4.

From Figure 5, it can be found that the SPA-based SVM model fits better at each temperature under different feature selection methods. According to Table 4, their $R_{p}^{2}$ values are above 0.95, and their RMSEP values are around 0.3. Meanwhile, the RPD is more stable between 1.6 and 1.8 for different temperatures. This indicates that the support vector machine model using linear kernel functions has an overall improvement in model performance at each temperature compared to PLS, thus suggesting that it has a better generalization capability and can be better adapted to the linearly differentiable case. For SVM modeling, the SPA-based method of selecting feature bands to detect soil nitrogen is the most effective. However, the RPD of both PLS and SVM models did not reach 2, so it could not be practically applied to the detection of soil nitrate–nitrogen.

#### 2.4.3. Prediction Model of Soil Nitrogen Content Based on Artificial Neural Network 

**Figure 6:**
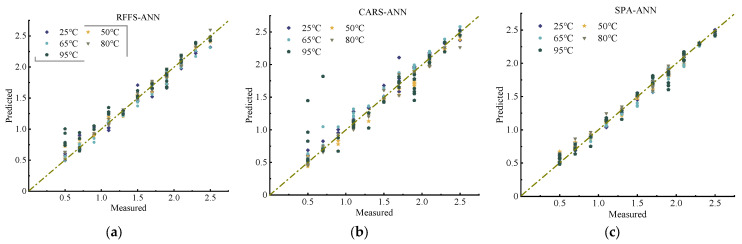
Relationship between actual and predicted values of soil nitrogen content based on the ANN model: (**a**) RFFS-ANN, (**b**) CARS-ANN, and (**c**) SPA-ANN.

The results according to ANN modeling are shown in Figure 6 and Table 5. The correlation coefficient values of the modeling of nitrogen predicted by the ANN algorithm are high overall, while the RMSEP values are low, and the relative analytical errors’ RPDs are stable—basically above 2—especially after drying at 80 °C, based on Table 5.

Combined with Figure 6, this shows that, as far as ANN modeling is concerned, the SPA-based method of selecting characteristic bands to detect soil nitrogen works best, indicating that the model can be used for quantitative predictive analysis. In general, the accuracy of the artificial neural network model was greatly compared with both the PLS and SVM models; thus, we may consider that there is still some nonlinear relationship between soil nitrogen and the selected sensitive waveform. The artificial neural network modeling approach can handle nonlinear relationships, meaning that it can better adapt to complex data and patterns [31]. ANN is also fault-tolerant, so even if some of the data or network nodes fail, it can still provide a useful result. Taken together, the modeling of soil spectra using the ANN algorithm can be used to predict soil nitrate–nitrogen content with good results in this study.

## 3. Discussion

### 3.1. Comparison of the Three Modeling Methods

**Figure 7:**
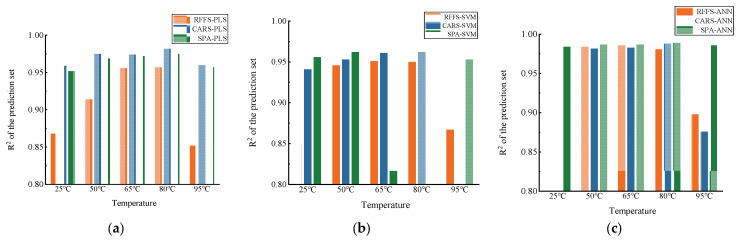
Histogram of actual and predicted values of soil N content for different models: (**a**) modeling results based on PLS, (**b**) modeling results based on SVM, and (**c**) modeling results based on ANN.

In this study, three modeling methods, namely PLS, SVM, and ANN, were used for modeling and analysis to investigate the effect of drying temperature on the detection of soil nitrate–nitrogen content via the use of NIR sensors. The comparison of the PLS model, SVM model, and ANN model based on different preprocessing methods is shown in Figure 7.

According to Figure 7, the prediction models of both PLS and SVM have high correlation coefficients but are unstable. Moreover, ANN is overall higher than both machine-learning models at each dry temperature. For instance, it can be seen that after drying at 80 °C, CARS-PLS and SPA-SVM both reach the highest correlation coefficients of the prediction set, 0.982 and 0.970, respectively, according to Table 3 and Table 4. Meanwhile, SPA-ANN has a high correlation coefficient of 0.989 at this temperature, according to Table 5. In short, different algorithms have different effects on soil nitrogen detection at the same temperature. The accuracy and stability of the ANN prediction model are better than that of the PLS and SVM models. The explanation for this may be that PLS can better handle the problem of multiple correlations of variables but not the nonlinear problem, and SVM can deal with the issue of nonlinear feature interactions, but sometimes it is hard to find a suitable kernel function. However, ANN, as a deep-learning model, has better adaptiveness and can automatically adjust the model parameters according to the changes in data. Moreover, it is robust to noise and outliers and can handle more complex data situations, so that the model accuracy can reach a high level [32]. In addition, although it performed best in this study, a large number of experiments are still needed to verify its stability as a measure of its application value, or a more simplified model can be continuously explored in subsequent studies.

### 3.2. Correlation Analysis of Soil-Drying Temperature and Model Accuracy

Based on some of the available studies, the soil-drying temperature has a significant effect on the detection of nitrogen in the NIR region. In this study, 11 different concentration gradients of nitrogen solutions were equipped and mixed evenly with soil; they were then placed at 25 °C for air-drying for 24 days, 50 °C for 36 h, 65 °C for 24 h, 80 °C for 18 h, and 95 °C for 12 h. This was followed by pressing the soil samples and collecting their spectral data for the modeling analysis.

**Figure 8:**
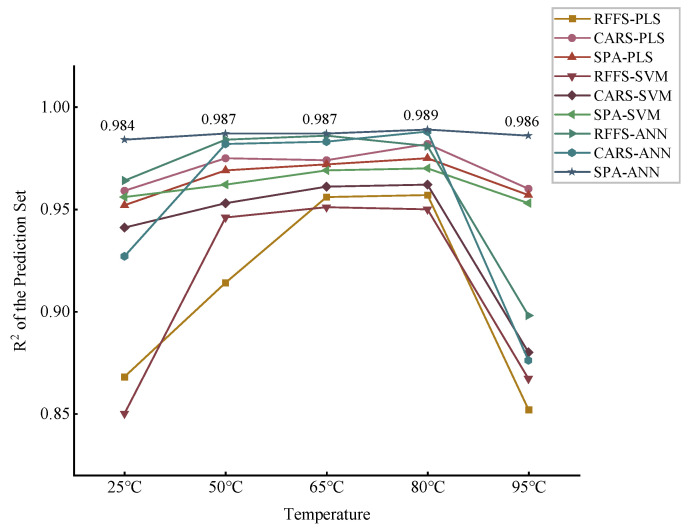
Comparison of modeling performance at different temperatures.

It can be seen first that the different modeling accuracies at varying temperatures are distinct, which is shown by the large gap between the algorithms at 25 °C and 95 °C, according to Figure 8. This shows that the drying temperature does have a great influence on the detection of soil nitrogen. Due to the influence of different temperatures and the differences in the principles of different feature selection methods, they pick out different feature bands combined with different modeling methods, resulting in different modeling effects.

Secondly, there is a gradual improvement in the overall performance of each model as the temperature increases. From 50 °C to 80 °C, the $R_{p}^{2}$ increased for most of the models. This is perhaps due to the fact that the effect of moisture gradually decreases with the increasing temperature. In addition, microbial activity at moderately dry temperatures may also lead to the nitrification of nitrogen compounds, thus causing nitrogen losses [33]. Whereas the greatest effect was observed at 25 °C, this may be due to the fact that low drying temperatures may not be able to completely dehydrate the soil. To the best of our knowledge, moisture also has a large effect on the NIR detection of soil nitrogen.

Finally, the high drying temperature is not conducive to the accurate NIR detection of soil nitrogen. A comprehensive comparison shows that the best performance was achieved at temperatures up to 80 °C. However, as the drying temperature increased from 80 °C to 95 °C, the performance of the models clearly decreased overall. This suggests that although the effect of moisture is completely eliminated at 95 °C, chemical processes such as thermal decomposition and volatilization may occur at higher temperatures [34]. In conclusion, appropriate drying temperatures can be of great help in the accurate detection of soil nitrogen by near-infrared.

## 4. Materials and Methods

### 4.1. Experiment Design

Soil samples were collected from Huai’an, Jiangsu Province, China, which is located between 32°43′00″–34°06′00″ N and 118°12′00″–119°36′30″ E. It is a transitional area between the southern warm temperate zone and the northern subtropical zone, with both northern and southern climatic characteristics. There are four distinct seasons: a warm and rainy spring, a hot and humid summer, a cool and dry autumn, and a cold and slightly rainy winter, with an average annual temperature of about 14 °C and annual precipitation of about 900 mm. Due to its flat topography and the influence of the Yangtze River basin and the Huai River basin, this area has a wide variety of soil types. The sandy loess used in this study is characterized by looseness, porosity, good permeability, and low water retention.

Soil samples were prepared as follows: Firstly, the collected soil was uniformly sieved and ground with a 60-mesh (about 0.25 mm) sieve under laboratory conditions, and a nitrogen solution containing 0.5–2.5 g/kg (with a concentration gradient of 0.2 g/kg) was prepared by mixing sodium–nitrate solution with distilled water. Next, the nitrogen solution was thoroughly mixed and blended with the soil samples. A total of 11 nitrogen concentrations were used, and 12 samples were replicated for each concentration gradient. Lastly, the experiments were conducted in five groups, and the drying conditions of the four groups of soil samples were 36 h at 50 °C, 24 h at 65 °C, 18 h at 80 °C, and 12 h at 95 °C to fully eliminate the effect of soil moisture on the detection of nitrogen in NIR. A control group was simultaneously set up, and the last group of soils was dried naturally at room temperature (i.e., 25 °C) for 24 days.

### 4.2. Spectrum Measurements

**Figure 9:**
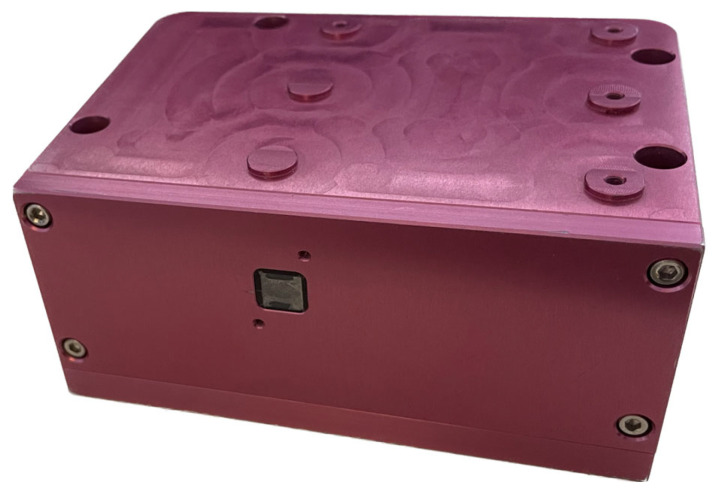
Experimental instrument—portable NIR spectrometer.

The NIR spectrometer used in this experiment was a reflection interferometer with two integrated tungsten–halogen lamps from Isuzu Optical Corporation (Shanghai, China). It can collect spectral information, including intensity, reflectance, and absorbance, in the range of 900–1700 nm. The device has an optical resolution of 10 nm and a signal-to-noise ratio of 5000:1 in a 1 s scan, and it has dimensions of 120 × 85 × 54 mm and weighs 900 g. The NIR spectrometer is shown in Figure 9. In this study, the spectral data were collected by using Isuzu Optics NIRez 2.0, a software for this spectroscopic instrument.

The device should be warmed up for 15 min before the spectroscopic measurement, and the calibration of the blackboard and whiteboard should be performed. To maintain the integrity of the original soil spectra and the speed of the detection process, the spectral acquisition parameters were set at 400 points, and three scans were set to use the average value as the spectral information of the samples. When scanning soil samples, the samples should be covered entirely with the light source window to avoid light leakage. It should be noted that its smooth side was chosen for collection to avoid the effects of cracks. In this study, the reflectance values of the spectra were recorded and used for subsequent modeling.

### 4.3. Spectral Analysis

Near-infrared light is a kind of electromagnetic wave between infrared and visible light. Its spectral information comes from the overlap of internal vibrations of O-H, C-H, N-H, and other hydrogen-containing groups with multi-frequencies and combined frequencies, which can be reflected in the characteristic signals of its spectral region of organic matter [35]. Furthermore, the spectral signal is stable and easy to obtain. According to Lambert’s absorption law, the spectral properties vary with the composition or structure of the material, and the spectra from different groups vary in regard to the position and intensity of the absorption peaks [36]. It is also affected by the nonuniform distribution of soil surface texture, density, and internal composition, making it difficult to eliminate all redundant information, such as overlap, from the spectral data [37,38,39]. Thus, to reduce spectral noise, baseline drift, and interference from other backgrounds, as well as to distinguish overlapping peaks for the purpose of qualitative or quantitative analysis of complex mixtures, five different preprocessing methods were used in this study to analyze the spectral information [40].

In this case, the Savitzky–Golay (S-G) smoothing algorithm uses a weighted average method to quantify the data in the moving window by polynomial least-squares fit and emphasizes the centrality of the centroid [41]. The basic idea of the multiplicative scattering correction (MSC) algorithm is to use the ideal spectrum to represent all spectra linearly regressed with the sample spectrum and to use the slope and intercept of the linear equation to correct the original spectrum [42]. The principle of the standard normal variation (SNV) algorithm is that the absorbance values at each wavelength point satisfy a certain distribution in each spectrum, and the spectral correction is performed according to this assumption [43]. The principle of the moving average smoothing (MA) algorithm is to filter out random noise from the original data by calculating the average of the samples and using it as a new data point. Wavelet transform (WT) can localize the analysis in time (space) frequency and gradually refine the signal (function) in multiple scales through the telescopic translation operation. Thus, the signal is decomposed into different frequency components. It makes the noise in the signal separate from the signal for denoising purposes [44,45,46].

### 4.4. Feature Band Selection Methods

NIR spectra can provide a large amount of spectral and spatial information related to soil nitrogen vitality properties. However, they also contain overlapping and redundant information, so it is necessary to use feature selection algorithms to obtain representative and important wavelengths to reduce irrelevant information and improve computational speed. By means of feature extraction techniques, the original high-dimensional spectral data are mapped or transformed to a lower dimensional space (while still retaining some of the necessary features of the original data), thus avoiding dimensional catastrophes to a large extent. This makes subsequent tasks, such as classification or clustering, not only more stable, efficient, and easy to handle but, more importantly, also yields better generalization performance [47]. In this study, the main data analysis software used was PyCharm 2023.1.2 (Community Edition) software.

#### 4.4.1. Random Forest Feature Selection Algorithm

The random forest feature selection (RFFS) method is based on the properties of the random forest algorithm (RF). The random forest algorithm is an integrated learning algorithm based on decision trees that are constructed to perform classification or regression by building multiple decision trees [48]. Moreover, feature selection is a randomized approach to split each node and, thus, compare the errors generated in different cases. That is, the Gini coefficient (gini) and out-of-bag (OOB) error rate are used as evaluation metrics to measure the contribution of different feature wavelengths. Next, the importance of different features can be calculated by taking the average values and sorting them in descending order. Then, based on this order, the corresponding proportion of feature wavelength variables is eliminated to obtain a new set of feature wavelengths. The above process is repeated on this basis until m features remain (m is a value set in advance). Finally, the error rate of each feature wavelength collection obtained in the above process is compared, and the feature spectral collection with the lowest error rate is selected as the best combination of feature wavelength variables [49].

#### 4.4.2. Competitive Adaptive Reweighted Sampling Algorithm

The competitive adaptive reweighted sampling (CARS) method is a feature variable selection method that combines Monte Carlo sampling with regression coefficients of the PLS model. It mimics the principle of “survival of the fittest” in Darwin’s theory [50]. The adaptive weighted sampling (ARS) algorithm combined with an exponential decay function is used in each sampling process. The PLS modeling retains the wavelength variables with the larger absolute weights of the regression coefficients and removes the wavelengths with the smaller weights of the regression coefficient values. The PLS modeling is then built based on the new subset. After several calculations, the subset of variables with the smallest root-mean-square error is selected based on ten-fold cross-validation. This subset contains the wavelength variables as the best combination of characteristic wavelength variables [51].

#### 4.4.3. Successive Projections Algorithm

The successive projections algorithm (SPA) is a forward variable selection algorithm for multivariate calibration to select the wavelength with the least redundancy [52]. It is based on the principle of first calculating the correlation between each feature variable and the target variable by calculating the correlation between each feature variable. Then, the target variables after the projection analysis are used to retain the most useful feature information. Specifically, it utilizes projection analysis of vectors by projecting wavelengths onto other wavelengths. The magnitudes of the projection vectors are then compared, and the wavelength with the largest projection vector is retained as the wavelength to be selected. Subsequently, based on the calibration model, the projected feature wavelengths are linearly combined to obtain the final feature spectral set and to realize the data dimensionality reduction [53]. SPA selects the combination of the near-infrared spectral variables with minimum redundancy and minimum covariance.

### 4.5. Model Evaluation Index

The regression model evaluates the detection performance of the established model by the coefficient of determination ($R^{2}$; Equation (1)), the root-mean-square error of prediction (RMSEP; Equation (2)), and the residual prediction deviation (RPD; Equation (3)). In this study, the coefficient of determination, $R^{2}$, reflects the ability of the constructed model to interpret the sample spectra, and RMSEP refers to the error between the predicted and actual nitrogen content. The RPD assesses the overall performance of the model. In general, the higher the $R^{2}$ and RPD and the lower the RMSEP, the better the performance of the prediction model. In this paper, $R_{c}^{2}$ and $R_{P}^{2}$ represent the correlation coefficients of the calibration and prediction sets, respectively, and RMSEC and RMSEP represent the root-mean-square error of the calibration and prediction sets, respectively. In addition, Mouazen et al. [54] classified the RPD values as follows. An RPD greater than 3 is a good quantitative forecast. Values between 2.5 and 3.0 mean that the model will have good predictive power. RPD values between 2.0 and 2.5 indicate that approximate quantitative prediction is possible. An RPD value between 1.5 and 2.0 means that the model is poor and needs to be improved. RPD values below 1.5 indicate that the model has poor predictive power and is not recommended.

(1) $$R^{2}=\sum_{i=1}^{n}{\left({\hat{y}}_{i}-\bar{y}\right)}^{2}/\sum_{i=1}^{n}{\left(y_{i}-\bar{y}\right)}^{2},$$

(2) $$RMSEP=\sqrt{\sum_{i=1}^{n}{\left({\hat{y}}_{i}-y_{i}\right)}^{2}/n},$$

(3) $$RPD=SD_{p}/RMSEP\sqrt{n/\left(n-1\right)},$$

where ${\hat{y}}_{i}$ is the predicted value, $\bar{y_{i}}\;$ is the mean of the observed value, $y_{i}$ is the observed value, and n is the number of predicted/observed values. $SD_{p}$ is the standard deviation of the prediction dataset.

## 5. Conclusions

The effects of drying temperature for the detection of soil nitrogen by near-infrared sensors were investigated. To begin with, the NIR reflectance spectral characteristics of soil samples at five different drying temperatures were explored, and then different preprocessing methods were applied to denoise the original real-time spectral signals of the soil. The modeling and analysis were made on the basis of PLS, and the best preprocessing method SG algorithm was selected to perform the smoothing of the spectra. Next, the RFFS, CARS, and SPA algorithms were selected to determine the characteristic bands, and the filtered reflectance spectra were used to establish the PLS model, SVM model, and ANN model for predicting soil nitrogen content. The findings provided a rapid, convenient, and environmentally friendly method for the real-time detection of soil nitrogen. They also provided a theoretical basis for the development of soil management and precision agriculture. The main conclusions are as follows.

(1) The analysis of soil reflectance spectral characteristics showed that the whole soil spectral curve shifted along the vertical direction with the change of drying temperature, which indicated that the varying of temperature and nitrate–nitrogen content of the drying soil would lead to a change in soil NIR reflectance. However, the spectral curved near 1400 nm at each drying temperature exhibited a very clear downward trend, indicating that hydrogen-containing groups of nitrogen, such as N-H, have stronger absorption in this band.
(2) PLS, SVM, and ANN regression models for predicting the soil nitrate–nitrogen content were developed using three feature selection algorithms, RFFS, CARS, and SPA, respectively. The results revealed that the PLS and SVM models could better estimate the soil nitrate–nitrogen concentration, but the accuracy and stability were inferior to that of the ANN model. Therefore, the authors concluded that they were not applicable to this study. The best accuracy of both the SPA-based ANN model and the highest correlation coefficient was reached at a drying temperature of 80 °C, indicating that the accuracy of ANN modeling based on deep learning was greatly improved and had a great advantage in predicting soil nitrate–nitrogen content in real-time.
(3) The soil-drying temperature has a significant effect on the detection of soil nitrate–nitrogen in NIR. As the drying temperature increased, the accuracy became better, while the accuracy dropped after the temperature reached 80 °C −95 °C, illustrating that high drying temperatures were not conducive to the NIR detection of soil nitrate–nitrogen. In summary, the selection of a suitable drying temperature was of great relevance to improve the accuracy of NIR detection of soil nitrogen. In future research, it may be possible to explore additional preprocessing algorithms and feature selection methods, as well as to investigate the effect of drying time in addition to temperature.

## Figures and Tables

**Table 1:** The prediction effects with different spectral pretreatments and different drying temperatures by using the partial least squares (PLS).

Methods	Group	Calibration Set		Prediction Set			N
		$R_{c}^{2}$	RMSEC (g/kg)	$R_{p}^{2}$	RMSEP (g/kg)	RPD	
RAW	25 °C	0.907	0.428	0.833	0.509	1.244	7
	50 °C	0.944	0.380	0.876	0.472	1.341	7
	65 °C	0.929	0.402	0.874	0.474	1.336	7
	80 °C	0.867	0.464	0.662	0.607	1.043	8
	95 °C	0.877	0.456	0.718	0.580	1.091	9
MA	25 °C	0.977	0.308	0.919	0.425	1.490	6
	50 °C	0.954	0.362	0.914	0.431	1.468	5
	65 °C	0.975	0.315	0.963	0.349	1.815	6
	80 °C	0.960	0.351	0.970	0.331	1.913	6
	95 °C	0.951	0.369	0.895	0.453	1.396	6
WT	25 °C	0.966	0.339	0.865	0.482	1.313	9
	50 °C	0.948	0.374	0.895	0.453	1.396	7
	65 °C	0.979	0.300	0.912	0.433	1.461	9
	80 °C	0.925	0.407	0.768	0.552	1.146	9
	95 °C	0.931	0.400	0.802	0.531	1.192	10
MSC	25 °C	0.976	0.311	0.881	0.467	1.354	10
	50 °C	0.985	0.275	0.937	0.399	1.587	10
	65 °C	0.985	0.276	0.920	0.423	1.495	10
	80 °C	0.929	0.403	0.723	0.577	1.096	9
	95 °C	0.932	0.398	0.733	0.572	1.107	10
SG	25 °C	0.959	0.353	0.941	0.392	1.615	5
	50 °C	0.976	0.311	0.973	0.322	1.967	6
	65 °C	0.977	0.306	0.964	0.347	1.824	5
	80 °C	0.975	0.312	0.977	0.309	2.05	7
	95 °C	0.964	0.343	0.951	0.375	1.689	7
SNV	25 °C	0.944	0.383	0.845	0.491	1.245	8
	50 °C	0.968	0.336	0.912	0.426	1.435	8
	65 °C	0.966	0.341	0.864	0.474	1.287	8
	80 °C	0.959	0.354	0.782	0.543	1.164	10
	95 °C	0.932	0.398	0.733	0.572	1.107	10

**Table 2:** Characteristic wavelength selection results based on RFFS, CARS, and SPA.

Methods	Temperature	Variable Number	Proportion
RFFS	25 °C	11	2.75%
	50 °C	31	7.75%
	65 °C	16	4%
	80 °C	28	7%
	95 °C	16	4%
CARS	25 °C	24	6%
	50 °C	24	6%
	65 °C	30	7.5%
	80 °C	30	7.5%
	95 °C	33	8.25%
SPA	25 °C	18	4.5%
	50 °C	8	2%
	65 °C	14	3.5%
	80 °C	14	3.5%
	95 °C	12	3%

**Table 3:** Results of PLS modeling based on different feature selection algorithms.

Methods	Temperature	$R_{c}^{2}$	RMSEC (g/kg)	$R_{p}^{2}$	RMSEP (g/kg)	RPD
RFFS-PLS	25 °C	0.847	0.479	0.868	0.479	1.320
	50 °C	0.932	0.401	0.914	0.423	1.444
	65 °C	0.971	0.326	0.956	0.364	1.737
	80 °C	0.976	0.313	0.957	0.356	1.716
	95 °C	0.896	0.439	0.852	0.493	1.283
CARS-PLS	25 °C	0.973	0.320	0.959	0.358	1.766
	50 °C	0.983	0.286	0.975	0.315	2.088
	65 °C	0.981	0.294	0.974	0.320	1.979
	80 °C	0.985	0.278	0.982	0.292	2.170
	95 °C	0.975	0.313	0.960	0.356	1.777
SPA-PLS	25 °C	0.972	0.321	0.952	0.372	1.700
	50 °C	0.967	0.335	0.969	0.334	1.896
	65 °C	0.981	0.292	0.972	0.326	1.939
	80 °C	0.984	0.284	0.975	0.312	1.959
	95 °C	0.976	0.336	0.957	0.362	1.750

**Table 4:** Results of building SVM models based on different feature selection algorithms.

Methods	Temperature	$R_{c}^{2}$	RMSEC (g/kg)	$R_{p}^{2}$	RMSEP (g/kg)	RPD
RFFS-SVM	25 °C	0.832	0.488	0.850	0.487	1.255
	50 °C	0.988	0.267	0.946	0.378	1.619
	65 °C	0.970	0.327	0.951	0.368	1.663
	80 °C	0.939	0.393	0.950	0.370	1.652
	95 °C	0.867	0.463	0.867	0.472	1.294
CARS-SVM	25 °C	0.949	0.378	0.941	0.385	1.587
	50 °C	0.969	0.343	0.953	0.365	1.675
	65 °C	0.976	0.308	0.961	0.348	1.755
	80 °C	0.976	0.311	0.962	0.346	1.767
	95 °C	0.838	0.463	0.880	0.460	1.330
SPA-SVM	25 °C	0.978	0.307	0.956	0.359	1.705
	50 °C	0.965	0.340	0.962	0.346	1.770
	65 °C	0.977	0.311	0.969	0.329	1.860
	80 °C	0.985	0.280	0.970	0.325	1.880
	95 °C	0.959	0.351	0.953	0.363	1.683

**Table 5:** Results of ANN models based on different feature selection algorithms.

Methods	Temperature	$R_{c}^{2}$	RMSEC (g/kg)	$R_{p}^{2}$	RMSEP (g/kg)	RPD
RFFS-ANN	25 °C	0.985	0.280	0.964	0.350	1.847
	50 °C	0.996	0.198	0.984	0.282	2.196
	65 °C	0.994	0.215	0.986	0.272	2.279
	80 °C	0.993	0.229	0.981	0.294	2.107
	95 °C	0.983	0.282	0.898	0.434	1.360
CARS-ANN	25 °C	0.984	0.291	0.927	0.407	1.502
	50 °C	0.990	0.249	0.982	0.287	2.128
	65 °C	0.997	0.181	0.983	0.280	2.181
	80 °C	0.998	0.166	0.988	0.260	2.378
	95 °C	0.993	0.224	0.876	0.467	1.325
SPA-ANN	25 °C	0.996	0.199	0.984	0.279	2.221
	50 °C	0.998	0.173	0.987	0.267	2.323
	65 °C	0.993	0.228	0.987	0.267	2.314
	80 °C	0.998	0.178	0.989	0.257	2.411
	95 °C	0.997	0.176	0.986	0.281	2.352

## Data Availability

Not applicable.

## Sample Availability

Not available.

## References

[1] Potdar R.P., Shirolkar M.M., Verma A.J., More P.S., Kulkarni A. (2021). Determination of soil nutrients (NPK) using optical methods: A mini review. J. Plant Nutr..

[2] Liu J., Cai H., Chen S., Pi J., Zhao L. (2023). A Review on Soil Nitrogen Sensing Technologies: Challenges, Progress and Perspectives. Agriculture.

[3] Ma X., Bifano L., Fischerauer G. (2023). Evaluation of Electrical Impedance Spectra by Long Short-Term Memory to Estimate Nitrate Concentrations in Soil. Sensors.

[4] Ward M., Jones R., Brender J., De Kok T., Weyer P., Nolan B., Villanueva C., Van Breda S. (2018). Drinking Water Nitrate and Human Health: An Updated Review. Int. J. Environ. Res. Public Health.

[5] Su R., Wu J., Hu J., Ma L., Ahmed S., Zhang Y., Abdulraheem M.I., Birech Z., Li L., Li C. (2022). Minimalizing Non-Point Source Pollution Using a Cooperative Ion-Selective Electrode System for Estimating Nitrate Nitrogen in Soil. Front. Plant Sci..

[6] Yu J., Yin X., Raper T.B., Jagadamma S., Chi D. (2019). Nitrogen Consumption and Productivity of Cotton under Sensor-based Variable-rate Nitrogen Fertilization. Agron. J..

[7] Qi J., Tian X., Li Y., Fan X., Yuan H., Zhao J., Jia H. (2020). Design and Experiment of a Subsoiling Variable Rate Fertilization Machine. Int. J. Agric. Biol. Eng..

[8] Nogueira Martins R., Magalhães Valente D.S., Fim Rosas J.T., Souza Santos F., Lima Dos Santos F.F., Nascimento M., Campana Nascimento A.C. (2020). Site-Specific Nutrient Management Zones in Soybean Field Using Multivariate Analysis: An Approach Based on Variable Rate Fertilization. Commun. Soil Sci. Plant Anal..

[9] Mouazen A.M., Kuang B. (2016). On-Line Visible and near Infrared Spectroscopy for in-Field Phosphorous Management. Soil Tillage Res..

[10] Xiao S., He Y. (2019). Application of Near-Infrared Spectroscopy and Multiple Spectral Algorithms to Explore the Effect of Soil Particle Sizes on Soil Nitrogen Detection. Molecules.

[11] Xu S., Wang M., Shi X., Yu Q., Zhang Z. (2021). Integrating Hyperspectral Imaging with Machine Learning Techniques for the High-Resolution Mapping of Soil Nitrogen Fractions in Soil Profiles. Sci. Total Environ..

[12] Zhang Y., Li M., Zheng L., Qin Q., Lee W.S. (2019). Spectral Features Extraction for Estimation of Soil Total Nitrogen Content Based on Modified Ant Colony Optimization Algorithm. Geoderma.

[13] Nocita M., Stevens A., Noon C., Van Wesemael B. (2013). Prediction of Soil Organic Carbon for Different Levels of Soil Moisture Using Vis-NIR Spectroscopy. Geoderma.

[14] Sorenson P.T., Quideau S.A., Rivard B. (2018). High Resolution Measurement of Soil Organic Carbon and Total Nitrogen with Laboratory Imaging Spectroscopy. Geoderma.

[15] Wang Y., Cui B., Zhou Y., Sun X., Li D., Zhao C. (2019). Advances in monitoring soil nutrients by near infrared spectroscopy. Computer and Computing Technologies in Agriculture XI.

[16] Li M. (2006). Spectral Analysis Technique and Its Application.

[17] Debaene G., Niedźwiecki J., Pecio A., Żurek A. (2014). Effect of the Number of Calibration Samples on the Prediction of Several Soil Properties at the Farm-Scale. Geoderma.

[18] Wang Y., Li M., Ji R., Wang M., Zheng L. (2021). A Deep Learning-Based Method for Screening Soil Total Nitrogen Characteristic Wavelengths. Comput. Electron. Agric..

[19] Xiao S., He Y., Dong T., Nie P. (2018). Spectral Analysis and Sensitive Waveband Determination Based on Nitrogen Detection of Different Soil Types Using Near Infrared Sensors. Sensors.

[20] Wang Q., Zhang H., Li F., Gu C., Qiao Y., Huang S. (2021). Assessment of Calibration Methods for Nitrogen Estimation in Wet and Dry Soil Samples with Different Wavelength Ranges Using Near-Infrared Spectroscopy. Comput. Electron. Agric..

[21] He Y., Xiao S., Nie P., Dong T., Qu F., Lin L. (2017). Research on the Optimum Water Content of Detecting Soil Nitrogen Using Near Infrared Sensor. Sensors.

[22] An X., Li M., Zheng L., Sun H. (2015). Eliminating the Interference of Soil Moisture and Particle Size on Predicting Soil Total Nitrogen Content Using a NIRS-Based Portable Detector. Comput. Electron. Agric..

[23] Zhou P., Yang W., Li M., Wang W. (2021). A New Coupled Elimination Method of Soil Moisture and Particle Size Interferences on Predicting Soil Total Nitrogen Concentration through Discrete NIR Spectral Band Data. Remote Sens..

[24] Nie P., Dong T., He Y., Xiao S., Qu F., Lin L. (2018). The Effects of Drying Temperature on Nitrogen Concentration Detection in Calcium Soil Studied by NIR Spectroscopy. Appl. Sci..

[25] Cartes P., Jara A.A., Demanet R., Mora M.D.L.L. (2009). Urease Activity and Nitrogen Mineralization Kinetics as Affected by Temperature and Urea Input Rate in Southern Chilean Andisols. Rev. Cienc. Suelo Nutr. Veg..

[26] Fystro G. (2002). The Prediction of C and N Content and Their Potential Mineralisation in Heterogeneous Soil Samples Using Vis–NIR Spectroscopy and Comparative Methods. Plant Soil.

[27] Stenberg B. (2010). Effects of Soil Sample Pretreatments and Standardised Rewetting as Interacted with Sand Classes on Vis-NIR Predictions of Clay and Soil Organic Carbon. Geoderma.

[28] Nie P., Dong T., He Y., Xiao S. (2018). Research on the Effects of Drying Temperature on Nitrogen Detection of Different Soil Types by Near Infrared Sensors. Sensors.

[29] Nie P., Dong T., He Y., Qu F. (2017). Detection of Soil Nitrogen Using Near Infrared Sensors Based on Soil Pretreatment and Algorithms. Sensors.

[30] Wang Y., Li M., Ji R., Wang M., Zheng L. (2020). Comparison of Soil Total Nitrogen Content Prediction Models Based on Vis-NIR Spectroscopy. Sensors.

[31] Solihat N.N., Son S., Williams E.K., Ricker M.C., Plante A.F., Kim S. (2022). Assessment of Artificial Neural Network to Identify Compositional Differences in Ultrahigh-Resolution Mass Spectra Acquired from Coal Mine Affected Soils. Talanta.

[32] Feng Y., Cui N., Hao W., Gao L., Gong D. (2019). Estimation of Soil Temperature from Meteorological Data Using Different Machine Learning Models. Geoderma.

[33] Dhar N.R. (1943). Improvement of the Nitrogen Status of Soils and the Origin of Soil Nitrogen. Nature.

[34] Dettmann U., Kraft N.N., Rech R., Heidkamp A., Tiemeyer B. (2021). Analysis of Peat Soil Organic Carbon, Total Nitrogen, Soil Water Content and Basal Respiration: Is There a ‘Best’ Drying Temperature?. Geoderma.

[35] Wang W., Yang W., Zhou P., Cui Y., Wang D., Li M. (2022). Development and Performance Test of a Vehicle-Mounted Total Nitrogen Content Prediction System Based on the Fusion of near-Infrared Spectroscopy and Image Information. Comput. Electron. Agric..

[36] Mouazen A.M., De Baerdemaeker J., Ramon H. (2005). Towards Development of On-Line Soil Moisture Content Sensor Using a Fibre-Type NIR Spectrophotometer. Soil Tillage Res..

[37] Zhang Z., Ding J., Zhu C., Wang J., Ma G., Ge X., Li Z., Han L. (2021). Strategies for the Efficient Estimation of Soil Organic Matter in Salt-Affected Soils through Vis-NIR Spectroscopy: Optimal Band Combination Algorithm and Spectral Degradation. Geoderma.

[38] Wu C., Zheng Y., Yang H., Yang Y., Wu Z. (2021). Effects of Different Particle Sizes on the Spectral Prediction of Soil Organic Matter. CATENA.

[39] Divya Y., Sanjeevi S., Ilamparuthi K. (2014). A Study on the Hyperspectral Signatures of Sandy Soils with Varying Texture and Water Content. Arab. J. Geosci..

[40] Gorry P.A. (1990). General Least-Squares Smoothing and Differentiation by the Convolution (Savitzky-Golay) Method. Anal. Chem..

[41] Chen J., Jönsson P., Tamura M., Gu Z., Matsushita B., Eklundh L. (2004). A Simple Method for Reconstructing a High-Quality NDVI Time-Series Data Set Based on the Savitzky–Golay Filter. Remote Sens. Environ..

[42] Isaksson T., Næs T. (1988). The Effect of Multiplicative Scatter Correction (MSC) and Linearity Improvement in NIR Spectroscopy. Appl. Spectrosc..

[43] Hsu H.-P., Binder K., Paul W. (2009). How to Define Variation of Physical Properties Normal to an Undulating One-Dimensional Object. Phys. Rev. Lett..

[44] Daubechies I. (1990). The Wavelet Transform, Time-Frequency Localization and Signal Analysis. IEEE Trans. Inf. Theory.

[45] Zhang Y., Li M., Zheng L., Zhao Y., Pei X. (2016). Soil Nitrogen Content Forecasting Based on Real-Time NIR Spectroscopy. Comput. Electron. Agric..

[46] Hutengs C., Ludwig B., Jung A., Eisele A., Vohland M. (2018). Comparison of Portable and Bench-Top Spectrometers for Mid-Infrared Diffuse Reflectance Measurements of Soils. Sensors.

[47] Vohland M., Ludwig M., Thiele-Bruhn S., Ludwig B. (2014). Determination of Soil Properties with Visible to Near- and Mid-Infrared Spectroscopy: Effects of Spectral Variable Selection. Geoderma.

[48] Breiman L. (2001). Random Forests. Mach. Learn..

[49] Xu Y., Bao S., Pradhan A.K. (2021). Modeling Drivers’ Reaction When Being Tailgated: A Random Forests Method. J. Saf. Res..

[50] Yun Y.-H., Wang W.-T., Deng B.-C., Lai G.-B., Liu X., Ren D.-B., Liang Y.-Z., Fan W., Xu Q.-S. (2015). Using Variable Combination Population Analysis for Variable Selection in Multivariate Calibration. Anal. Chim. Acta.

[51] Krakowska B., Custers D., Deconinck E., Daszykowski M. (2016). The Monte Carlo Validation Framework for the Discriminant Partial Least Squares Model Extended with Variable Selection Methods Applied to Authenticity Studies of Viagra^®^ Based on Chromatographic Impurity Profiles. Analyst.

[52] Yang H., Kuang B., Mouazen A.M. (2012). Quantitative Analysis of Soil Nitrogen and Carbon at a Farm Scale Using Visible and near Infrared Spectroscopy Coupled with Wavelength Reduction. Eur. J. Soil Sci..

[53] Araújo M.C.U., Saldanha T.C.B., Galvão R.K.H., Yoneyama T., Chame H.C., Visani V. (2001). The Successive Projections Algorithm for Variable Selection in Spectroscopic Multicomponent Analysis. Chemom. Intell. Lab. Syst..

[54] Mouazen A.M., De Baerdemaeker J., Ramon H. (2006). Effect of Wavelength Range on the Measurement Accuracy of Some Selected Soil Constituents Using Visual-Near Infrared Spectroscopy. J. Infrared Spectrosc..

